# Unusual Presentation of Dengue Fever Leading to Unnecessary Appendectomy

**DOI:** 10.1155/2015/465238

**Published:** 2015-06-17

**Authors:** Lovekesh Kumar, Mahendra Singh, Ashish Saxena, Yuvraj Kolhe, Snehal K. Karande, Narendra Singh, P. Venkatesh, Rambabu Meena

**Affiliations:** ^1^Department of Surgery, Hindu Rao Hospital, Delhi, India; ^2^Department of Surgery, AIIMS, Jodhpur 342005, India

## Abstract

Dengue fever is the most important arbovirus illness with an estimated incidence of 50–100 million cases per year. The common symptoms of dengue include fever, rash, malaise, nausea, vomiting, and musculoskeletal pain. Dengue fever may present as acute abdomen leading to diagnostic dilemma. The acute surgical complications of dengue fever include acute pancreatitis, acute acalculous cholecystitis, nonspecific peritonitis, and acute appendicitis. We report a case of dengue fever that mimicked acute appendicitis leading to unnecessary appendectomy. A careful history examination for dengue-related signs, and serial hemogram over the first 3-4 days of disease may prevent unnecessary appendectomy.

## 1. Introduction

Dengue fever is the most important arbovirus illness worldwide with an estimated incidence of 50–100 million cases per year [1]. In India, it is one of the most common mosquito-borne diseases seen during the rainy season when the “*Aedes*” mosquitos breed [2]. The common symptoms of dengue include fever, rash, malaise, nausea, vomiting, and musculoskeletal pain [2]. Though being generally a mild self-limiting disease, about one-third of patients develop severe complications including dengue hemorrhagic fever (DHF) and dengue shock syndrome (DSS) [2, 3]. Few reports of uncommon complications such as acute myocarditis, acute hepatic failure, acalculous cholecystitis, and acute pancreatitis have been reported [4, 5]. In addition, cases have been reported on dengue fever mimicking acute appendicitis. Careful evaluation of dengue fever in patients presenting with signs and symptoms mimicking acute appendicitis in dengue endemic area will avoid unnecessary appendectomy [4, 5]. We report a case of dengue fever that mimicked acute appendicitis leading to unnecessary appendectomy.

## 2. Presentation of Case

A 17-year-old male presented to the surgical emergency unit with history of abdominal pain for 2 days that began in the periumbilical area but later it was localized to the right iliac fossa. There was associated history of high grade fever, anorexia, and several episodes of vomiting for 2 days. There was no history of diarrhoea, musculoskeletal pain, and petechial rash. He had no significant past medical history and was not taking any medications.

General physical examination revealed a temperature of 100°F, tachycardia of 122/minute, normal respiratory rate of 16/minute, and normal blood pressure of 118/70 mmhg. Examination of the abdomen revealed marked tenderness in the right iliac fossa. There was no guarding or rigidity on palpation of abdomen. Complete hemogram revealed thrombocytopenia (platelet count 102000/mm^3^). The rest of routine blood investigations including haemoglobin (14 gm/dL), hematocrit (51%), and leukocyte (5,500/mm^3^) counts were in normal range. Patient underwent abdominal ultrasound in view of acute appendicitis but it was not helpful as it showed only minimal fluid collection in right iliac fossa and appendix was not visualized. The rest of ultrasonic findings and plain X-ray of abdomen were in normal range.

The patient was kept under observation providing adequate analgesia but on reassessment after 12 hours there was worsening of right iliac fossa pain. As history and clinical features were in favour of acute appendicitis, a diagnosis of acute appendicitis was made. Due to unavailability of diagnostic laparoscopy in our emergency set-up at that time, an open appendectomy was performed via a Lanz incision. On exploration, a normal appendix was found with multiple small mesenteric lymph nodes. Further exploration of bowel was done to rule out Meckel's diverticulum but it was not found. Histopathological examination of resected appendix was suggestive of normal appendix. In postoperative period, patient was still running fever and was complaining of musculoskeletal pain. Repeat blood investigations were done which showed thrombocytopenia (65000/mm^3^) and leucopenia (3,600/mm^3^). Dengue fever was suspected and was confirmed with a positive commercial IgG and IgM enzyme-linked immunoabsorbent assay.

Patient was successfully managed conservatively with adequate analgesics, bed rest, and fluid therapy. Repeat routine blood investigations after eight days were in normal range. During the follow-up period of four weeks, he was completely recovered from illness.

## 3. Discussion

Dengue fever is common in tropical countries including India with frequent outbreaks in rainy season [2]. The virus is having four serotypes (DEN1–DEN4) and is transmitted between humans by “*Aedes aegypti*” and “*Aedes albopictus*” [4]. Dengue fever usually presents as an acute febrile illness, musculoskeletal pain, nausea, vomiting, and petechial rash [3, 4]. Dengue fever may present as acute abdomen leading to diagnostic dilemma. The acute surgical complications of dengue fever include acute pancreatitis, acute acalculous cholecystitis, nonspecific peritonitis, and acute appendicitis [6–8].

The incidence of acute abdominal signs reported in dengue fever has ranged from 4.3% to 12.04% [5, 9]. Khor et al. reviewed 328 patients with DHF/DSS in a dengue epidemic area in southern Taiwan. They found that out of 328 patients with DHF/DSS only 14 had acute abdomen. Cause of acute abdomen was acute cholecystitis (6 acalculous and 4 calculous cholecystitis) in 10 patients, nonspecific peritonitis in 3 patients, and acute appendicitis in only 1 patient [5]. Shamim reviewed 357 patients with dengue fever and reported that 276 patients had nonspecific abdominal pain without overt abdominal signs, 43 (12.04%) had acute abdominal pain with definite abdominal signs, and only 38 (10.64%) presented without abdominal pain [9].

Premaratna et al. reported 12 cases of dengue fever mimicking acute appendicitis. On admission, ten out of twelve patients were having thrombocytopenia and eight of twelve patients were having leucopenia. Although thrombocytopenia and leukopenia are not diagnostic of dengue fever, they can help in making a diagnosis of dengue in a patient presenting with acute abdomen, during a dengue epidemic. Most of these patients develop leukopenia by the third day of illness [8]. Our patient presented with thrombocytopenia and later also developed leucopenia.

The reason for dengue fever presenting with acute abdomen is unclear. Enlarged mesenteric lymph nodes with serous fluid collection and oedema may present in dengue fever with acute abdomen, which are suggestive of inflammatory pathology [4, 8]. Cholestasis with gallbladder distension and cystic duct spasm have been suggested as possible causes of acalculous cholecystitis in dengue fever. Lymphoid hyperplasia and mesenteric adenitis may be possible underlying pathologies which mimic acute appendicitis in dengue fever [4, 8]. Enlarged mesenteric lymph nodes suggestive of mesenteric adenitis may explain symptoms and signs mimicking acute appendicitis in our patient. Shamim et al. reported that DHF and DSS usually present with acute abdomen [9]. Other theories proposed are plasma leakage from damaged capillary endothelium and high protein effusions with lymphocytic infiltrations in patients presenting with acute abdomen in dengue [5, 10].

The diagnosis of acute appendicitis is mainly dependent on clinical parameters. Usually patients presenting with acute appendicitis have a higher leukocyte count but a normal leukocyte count certainly does not rule out appendicitis. Therefore, patients presenting with clinical features suggestive of acute appendicitis and a normal leukocyte count need a careful approach in dengue epidemic area [8]. Abdominal signs and symptoms in dengue fever usually improved over 48–72 hours, so confirmation of dengue fever with dengue IgM serology and regular assessment of vitals, leukocyte counts, and platelet counts can be helpful in suspicious cases [8]. Some laboratory tests such as serological test, reverse transcription PCR, and dengue NS1 antigen can be helpful in diagnosis of dengue fever [11]. The dengue IgM and dengue IgG are the most commonly used serological tests. The criteria for positive dengue fever are positive dengue IgM and fourfold rise in dengue IgG. Accuracy of these tests is dependent on the timing of their sample collection. Dengue IgM usually rose earlier during days 3–5 of illness and will be higher in primary infection than in secondary infection, whereas the rise in IgG is delayed during days 5–10 of illness [11]. Since these serological tests might not provide an early diagnosis, dengue NS1 (nonstructural protein 1) antigen detection can be used for early diagnosis [12]. Detection of NS1 antigen has moderately high sensitivity and very high specificity to dengue infection. The combination of NS1 antigen and IgM assay would further increase the sensitivity in suspicious cases [12]. Conservative trial can be given for one or two days if these tests are positive and CT abdomen can be used for further evaluation in case of worsening abdominal symptoms.

Diagnosis of dengue in most of the cases can be made by suggestive clinical features, but in our case we were misled as the initial presentation was of acute abdomen. So it is important that surgeon in dengue endemic areas be aware of this overlapping presentation in order to prevent unnecessary surgery-related morbidity or even mortality.
